# Retrospective study testing next generation sequencing of selected cancer-associated genes in resected prostate cancer

**DOI:** 10.18632/oncotarget.7343

**Published:** 2016-02-12

**Authors:** Marco Lo Iacono, Consuelo Buttigliero, Valentina Monica, Enrico Bollito, Diletta Garrou, Susanna Cappia, Ida Rapa, Francesca Vignani, Valentina Bertaglia, Cristian Fiori, Mauro Papotti, Marco Volante, Giorgio V. Scagliotti, Francesco Porpiglia, Marcello Tucci

**Affiliations:** ^1^ University of Turin, Department of Oncology, Orbassano, Italy

**Keywords:** next generation sequencing, prostate cancer, precision medicine, genetic characterization, prognostic factors

## Abstract

**Purpose:**

Prostate cancer (PCa) has a highly heterogeneous outcome. Beyond Gleason Score, Prostate Serum Antigen and tumor stage, nowadays there are no biological prognostic factors to discriminate between indolent and aggressive tumors.

The most common known genomic alterations are the TMPRSS-ETS translocation and mutations in the PI3K, MAPK pathways and in p53, RB and c-MYC genes.

The aim of this retrospective study was to identify by next generation sequencing the most frequent genetic variations (GVs) in localized and locally advanced PCa underwent prostatectomy and to investigate their correlation with clinical-pathological variables and disease progression.

**Results:**

Identified non-synonymous GVs included TP53 p.P72R (78% of tumors), two CSFR1 SNPs, rs2066934 and rs2066933 (70%), KDR p.Q472H (67%), KIT p.M541L (28%), PIK3CA p.I391M (19%), MET p.V378I (10%) and FGFR3 p.F384L/p.F386L (8%). TP53 p.P72R, MET p.V378I and CSFR1 SNPs were significantly associated with the HI risk group, TP53 and MET variations with T≥T2c. FGFR3 p.F384L/p.F386L was correlated with T≤T2b. MET p.V378I mutation, detected in 20% of HI risk patients, was associated with early biochemical recurrence.

**Experimental design:**

Nucleic acids were obtained from tissue samples of 30 high (HI) and 30 low-intermediate (LM) risk patients, according to D'Amico criteria. Genomic DNA was explored with the Ion_AmpliSeq_Cancer_Hotspot_Panel_v.2 including 50 cancer-associated genes. GVs with allelic frequency (AF) ≥10%, affecting protein function or previously associated with cancer, were correlated with clinical-pathological variables.

**Conclusion:**

Our results confirm a complex mutational profile in PCa, supporting the involvement of TP53, MET, FGFR3, CSF1R GVs in tumor progression and aggressiveness.

## INTRODUCTION

Prostate cancer (PCa) is the most common type of cancer in men, with nearly 220,000 new cases expected in 2015 in USA [1]. The widespread use of serum prostate specific antigen (PSA) as a screening tool led to the increased frequency of PCa diagnoses. A subgroup of patients with extremely localized PCa may be potentially over-treated because only a proportion will develop a clinically detectable disease during their lifespan. PCa is extremely heterogeneous, ranging from a chronic indolent illness to an aggressive rapidly fatal malignancy. Beyond the classical prognostic factors of tumor stage (T), PSA level and Gleason score (GS), to date there are no biological prognostic factors able to discriminate between indolent and aggressive tumors. The abovementioned clinical prognostic factors allow clinicians to categorize patients into broad risk groups (low, intermediate and high risk) but they incompletely explain the observed heterogeneity of clinical outcome [2]. Moreover, specific pathological assessments as, for instance, the GS is operator-dependent and inter-observer variability has been reported. Therefore, new prognosticators are needed to improve clinician's skill in predicting individual likelihood of each PCa to progress and metastasize.

Advances in sequencing technologies generated a huge amount of data about the mutational events underlying development, progression and treatment response in cancer. Even if the spectrum of genetic alterations in PCa is heterogeneous, these mutations are more frequently reported in the PI3K and MAPK pathways and in p53, RB and c-MYC genes, all known to affect tumorigenesis in a wide spectrum of tumors, while there are others genetic alterations more specifically reported in PCa [3]. The vast majority of PCa harbors ETS rearrangements, generally as TMPRSS-ERG fusion [4]. In addition, PTEN and TP53 tumor suppressor genes are deleted in about 20-40% of PCa while Speckle-Type POZ Protein mutations occur in about 10% of the samples [5, 6].

In this retrospective study, formalin-fixed, paraffin-embedded (FFPE), prostatectomy specimens from 60 patients with localized or locally advanced PCa having clinical annotates were collected. Tumor samples were analyzed by next generation sequencing (NGS) in order to test the most frequent genetic mutations reported in cancers and to investigate their potential correlation with clinical pathological variables and disease progression.

## RESULTS

Sixty cases of localized or locally advanced PCa submitted to radical prostatectomy at San Luigi Gonzaga Hospital (Orbassano, Turin) were retrospectively extracted from an institutional clinical database. Based on PSA, GS and T, using the D'Amico criteria, tumors were categorized as high risk (HI, PSA>20 ng/ml, GS>7, T2c-T3, n=30) or low-intermediate risk (LM, low risk: PSA<10 ng/ml, GS <7, T1-T2a; intermediate risk: PSA: 10-20 ng/ml, GS=7, T2b, n=30).

Patients' characteristics are shown in Table 1. Median age was 66 (range: 49-76), median PSA before prostatectomy was 7 ng/ml (range: 3.2-21); 29 patients (48.3%) had GS=6, 28 (46.6%) GS=7 and 3 (5.1%) had GS=8. TMPRSS2:ERG fusion was found in 14/29 (48%) HI patients and in 5/26 LM (19%). AR expression was detectable in all samples analyzed but was not statistically different between two patient groups (p=0.9).

**Table 1:** Clinical pathological features in high and low/intermediate risk prostate cancer patients

		Tot N (%)	High (30)	Low/Med (30)	P value Fisher Test
**Age (median: 66 Years)**					<0.01
	Under	30 (50)	9	21	
	Over	30 (50)	21	9	
**PSA at diagnosis**					0.02
	≤10	46 (76.6)	18	28	(>10 ng)
	10-20	10 (16.7)	8	2	
	>20	4 (6.7)	1		
**PSA post prostatectomy**					0.37
	<0.02	50 (83.3)	23	27	
	≥0.02	10 (16.7)	7	3	
**Gleason Score**					≪.01
	<7	28 (46.6)	6	22	(≥7)
	=7	29 (48.3)	21	8	
	>7	3 (5.1)	3	0	
**Tumor Size (T)**					≪0.01
	T1-T2a	16 (26.6)	0	16	(≥2c)
	T2b	14 (23.3)	1	13	
	T2c-T3	30 (50)	29	1	
**Perineural invasion**					0.012
	no	10 (16.7)	1	9	
	yes	50 (83.3)	29	21	
**Vascular invasion**					0.03
	no	43 (71.7)	18	27	
	yes	13 (21.7)	10	3	
	missing	4 (6.6)			
**Surgical margins**					1
	R0	53 (88.3)	26	27	
	R1	7 (11.7)	4	3	
**Pelvic Lymphadenectomy**					≪0.01
	no	38 (63.3)	10	28	
	yes	22 (36.7)	20	2	
**Biochemical recurrence**					<0.01
	no	45 (75)	17	28	
	yes	15 (25)	13	2	
**ERG:TMPRSS2**					0.03
	no	41 (68.3)	16	25	
	yes	19 (31.7)	14	5	

Older age (> median), PSA at diagnosis > 10ng, Gleason Score ≥7, T≥2c, perineural/vascular invasion, pelvic lymphadenectomy, biochemical recurrence and TMPRSS2:ERG translocation were significantly associated with the HI group (Table 1). Gleason Score ≥7, T≥2c, vascular invasion, pelvic lymphadenectomy and PSA after prostatectomy >0.02 ng were significantly associated with early progression time. Gleason Score, vascular invasion and PSA after prostatectomy were independent predictors of early progression time (Gleason Score: p=0.02, HR=5.4 95% CI [1.24-23.37], vascular invasion: p=0.045, HR 3.86 95% CI [1.03-14.5], PSA after prostatectomy: p≪0.01, HR 121.4 95% CI [17.41-847.31]).

### CHP2 genetic profile

**Figure 1:**
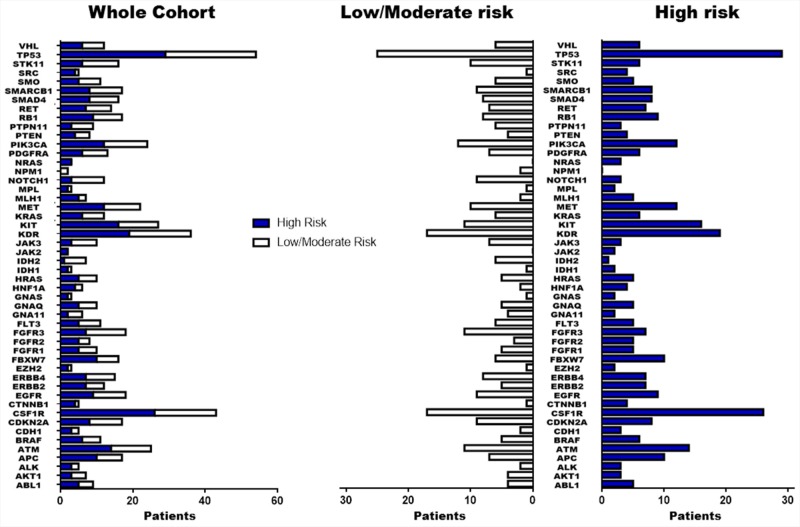
Summary of genetic variations identified by NGS Non synonymous/regulative variations identified in the 50 cancer-associated genes with at least 10% of allelic frequency are summarized in figure. The blue blocks indicate patients with High risk of PCa-recurrence while the white blocks those patients with low/moderate risk.

DNA was successfully extracted, amplified and sequenced from all samples. Non-synonymous genetic variations (GVs) with allelic frequency (AF) ≥10% are reported in Figure 1 and complete information for all genes included in analysis are presented in Supplementary Table S1. The most frequent non-synonymous GVs identified were TP53 p.P72R (COSM250061) in 78% of tumors, followed by two SNPs (rs2066934, rs2066933), within the 3′UTR of the CSFR1 gene in 70% of tumors, KDR p.Q472H (COSM149673) in 67%, KIT p.M541L (COSM28026) in 28%, PIK3CA p.I391M (COSM328028) in 19%, MET p.V378I (COSM3411512) in 10% and FGFR3 p.F384L/p.F386L (COSM724, COSM1539830) in 8%. The GVs at TP53 p.P72R, CSFR1 (rs2066934, rs2066933) and MET p.V378I were significantly associated with the HI risk group (p=0.027, p=0.047 and p=0.011, respectively). Moreover, TP53 p.P72R and MET p.V378I were also correlated to T≥T2c (p=0.032 and p=0.047, respectively). Conversely, genetic variation FGFR3 p.F384L/p.F386L was correlated with T≤T2b (p=0.004) and KDR p.Q472H with tumors lacking vascular invasion (p=0.021). Among synonymous GVs only the FGFR3 SNP rs3135898, found in 11 patients (18%), was significantly correlated to the HI group and T≥T2c tumors (p=0.042 and p=0.029, respectively). MET p.V378I mutation, clustered among 6 patients of the HI risk group (20%), was correlated to early PCa recurrence (p=0.02, HR 3.54 95% CI [1.38-44.98]). At the multivariable analysis, this mutation was an independent predictor of early recurrence of borderline statistical significance (p=0.055, HR 5.76 95% CI [0.97-34.31]) (Table 2).

**Table 2:** Main genetic variations identified in prostate cancer patients

Gene	Chr	Position	Ref	Alt	Type	AAChange	SNP ID	COSMIC ID	Patients affected	Correlation
**CSF1R**	chr5	149433596	T	G	UTR3	NA	rs2066934	NA	42	High Risk
**CSF1R**	chr5	149433597	G	A	UTR3	NA	rs2066933	NA	42	High Risk
**FGFR3**	chr4	1806131	T	C	Non-Synonymous	p.F384L, p.F386L	rs17881656	COSM724, COSM1539830	5	T≤2b
**FGFR3**	chr4	1807922	G	A	intronic	NA	rs3135898	NA	11	High Risk, T≥2c
**KDR**	chr4	55972974	T	A	Non-Synonymous	p.Q472H	rs1870377	COSM149673	28	Less invasive tumor
**MET**	chr7	116340270	G	A	Non-Synonymous	p.V378I	NA	COSM3411512	6	High Risk, T≥2c, short recurrence time
**TP53**	chr17	7579472	G	C	Non-Synonymous	p.P72R	rs1042522	COSM250061	47	High Risk, T≥2c

**Figure 2:**
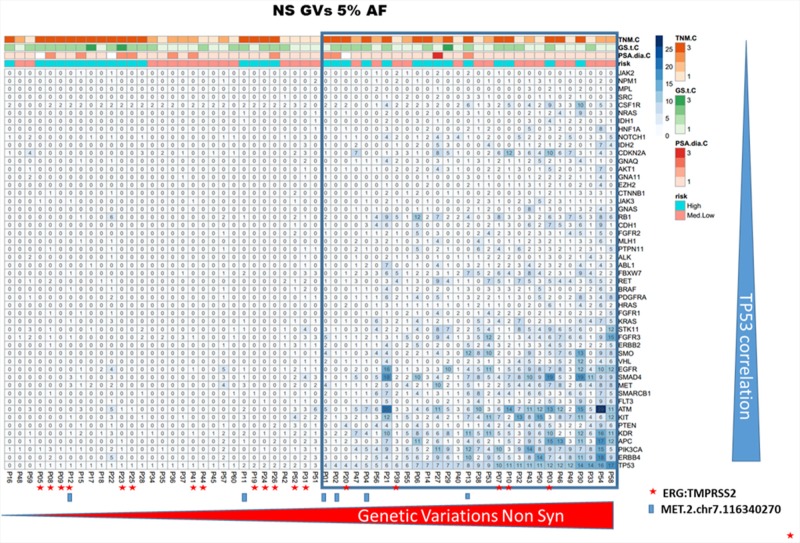
CHP2 genetic variability in prostate cancer The panel shows the heatmap of PCa samples, including only the non synonymous/regulative variations at ≥ 5% AF. The ≥ 5% filter was used only for graphic display. The blue tiles identify variations and color intensity is proportional to the number of variations observed in each PCa sample and indicated within the tile. Independently by the risk class, the group of cases having several GVs in many genes was associated with high TP53 mutational rate. Furthermore, only in a small subgroup of these high-mutated patients, the TMPRSS2-ERG translocation was detected (red star).

**Figure 3:**
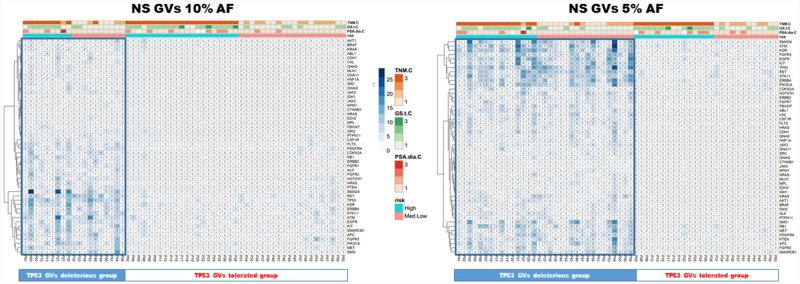
TP53 GVs deleterious correlate with high mutation rate patients Both heatmaps represent PCa patients with only the non synonymous/regulative variations at ≥ 10% (left panel) or ≥ 5% AF (right panel). Blue tiles contain the number of variations identified, visualized by proportional color intensity, observed in each PCa sample. The entire group of patients was clustered for “deleterious” or “tolerated” TP53 mutations, using a combination of PolyPhen-2 and SIFT software. In both panels, representing the applied AF filters of ≥10% and the ≥5%, respectively, patients with a high mutation rate were often included in “TP53 GVs deleterious group”.

Two groups of tumors were identified according to the CHP2 mutational profile findings: tumors with few non-synonymous GVs and tumors with several GVs in many genes. Independently from the risk class, the second group was associated with high TP53 mutational rate. To assess the robustness of this preliminary observation, the entire group of patients was clustered for “deleterious” or “tolerated” TP53 mutations, using a combination of PolyPhen-2 and SIFT software. These bioinformatic tools apply statistical algorithms based on sequence- and structure- information to predict the effect of GVs on proteins functionality/activity. As shown in Figure 2 and 3, both using the ≥10% and the ≥5% AF filter, patients with a high mutation rate were often included in “TP53 GVs deleterious group” and only few of these tumors showed TMPRSS2:ERG translocation.

**Figure 4:**
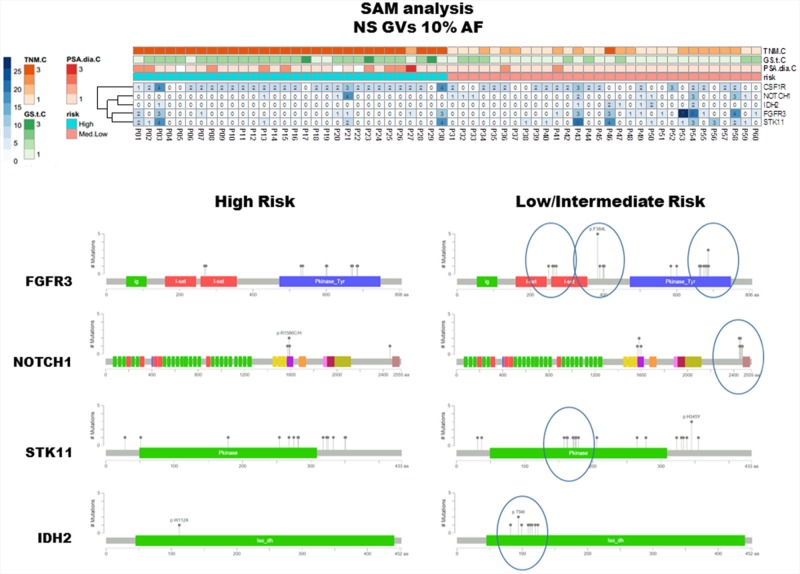
SAM analysis and protein domains localization of detected GVs The upper panel summarizes the results elaborated by means of SAM software and visualized through heatmap including non synonymous/regulative variations at ≥ 10% AF. CSF1R GVs were mainly identified in HI risk patients, in contrast NOTCH1, IDH2, FGFR3 and STK11 GVs were often observed in LM group. The MutationMapper software, which identifies protein regions affected by corresponding GVs, reflects the protein localization of such non-synonymous GVs, being enriched, in LM cluster, in limited and specific domains (lower right panel).

To highlight differences between HI and LM groups, the matrix of non-synonymous GVs (AF ≥10%) was also assessed by means of SAM-sequencing tools. This software allows detecting differences between two or more groups determining a false discovery rate (FDR) to increase robustness of data analyses. CSF1R GVs were associated to HI risk group and NOTCH1, IDH2, FGFR3 and STK11 often altered in LM groups (Figure 4, upper panel). Interestingly, some of these non-synonymous GVs were enriched in limited gene regions corresponding to specific protein domain (Figure 4, lower panel) [7]. GVs for the FGFR3 gene were consistently clustered in *I-set* domain, *P-kinase* Domain and between these two regions, as well as at the end of coding sequence for NOTCH1, in the middle of STK11 kinase domain and in IDH2 *iso_dh* domain.

To assess the expression pattern of CHP2 genes the NGS RNA-sequencing analysis was tested. After normalization by AmpliSeqRNA plugin, 22 valid expression profiles for the HI and 29 for the LM group were obtained. SAM-sequencing software identified, between the HI and the LM groups, differentially expressed transcripts for FBXW7, JAK2, GNAQ (t-test p=0.001, p=0.02 and p=0.02, respectively). No correlation between CHP2 RNA expression and clinical-pathological characteristics was identified. An association between TP53 mRNA low levels and “TP53 altered group” ≥5% AF was observed (p=0.02).

## DISCUSSION

In the last 10 years, NGS boosted biological and biomedical knowledge facilitating multi-gene mutational profiling using extremely small amounts of DNA, including that obtained by FFPE. The expanding application of NGS techniques has the potential for accurately mapping the type and extent of gene mutations in several solid tumors including PCa. In this retrospective study, the feasibility of assessing mutational changes by NGS using FFPE tissue samples was investigated using a commercially available panel of key cancer-associated genes. Despite the limited number of samples included in this study, some peculiarities in PCa GVs profile were identified. An enrichment for GVs in a quite restricted number of genes including TP53, CSF1R, KDR, KIT, ATM, PIK3CA, MET, EGFR, FGFR3, ranging from 90% to 30% of the analyzed samples, were documented and most of these genes were previously associated with PCa.

Genetic alterations in TP53 were the most common GVs and this observation agrees with previously published data that since early ‘90s documented TP53 mutations in PCa [8, 9]. Irrespective of risk stratification, we identified several alterations in this gene, some of which are already cancer-related and annotated in COSMIC database (Supplementary Table S1). In addition, patients with CHP2 high mutation rate display simultaneous “deleterious” TP53 mutations (Figure 2 and 3) and/or significant low levels of TP53 gene expression (p=0.02, GVs ≥5% AF). These data support the hypothesis of a reduced activity/expression of TP53 protein in PCa and suggest for its role in PCa tumorigenesis or tumor progression, as already suggested by others [9, 10]. High allelic variations at TP53 p.P72R, already identified in malignant pleural mesothelioma [11], were observed in PCa mainly in the HI risk group, suggesting its potential role as a risk factor. In agreement with our data, Zhang et al. showed that Caucasians with the Arg allele have an increased PCa risk [12]. A significant difference in the frequency of TP53 codon 72 variants between sporadic PCa and benign prostate hyperplasia was recently observed, implying that this polymorphism may have a role in tumor development [13]. In our study, we identified two SNPs in CSF1R gene, rs2066934 and rs2066933, mainly detected in HI risk group. Although these SNPs have been also recently reported in malignant pleural mesothelioma [11], scant information is available about these GVs. These two nucleotides trigger the 3′UTR region in a miRNA seed-complementary sequence, which could modulate response to imatinib by increasing CSF1R gene expression [14]. Two other studies which extensively explored NGS in advanced, castration resistant PCa did not detect any GVs in the CSF1R gene but differences in the disease setting and the NGS platforms used preclude any comparison [5, 6].

KIT p.M541L mutation, identified in about 30% of the tumors and without any relationship with the risk class, was already associated to clinical response to imatinib. In patients with aggressive fibromatosis harboring this mutation, a higher sensitivity to imatinib was initially observed but not confirmed in a subsequent study, possibly as the consequence of the rarity of this mutation in these rare tumors [15, 16]. Patients with chronic eosinophilic leukemia positive for KIT p.M541L showed clinical response to low dose imatinib [17]. In preclinical studies in PCa cells imatinib, alone or in combination with other cytotoxic therapies, showed a significant treatment effect [18–20]. Conversely, in clinical studies in patients with castration resistant PCa imatinib demonstrated only a modest activity [21–23]. These conflicting data between preclinical and clinical studies in PCa may be the result of a differential role of the CSF1R/KIT genetic alterations in patients and in cell lines. Our findings suggest additional studies testing the role of imatinib in genetically selected PCa patients.

The MET p.V378I mutation was detected in 6 tumors of the HI risk group and associated with early PCa recurrence following radical surgery (Table 2). Whereas the proto-oncogene MET has been associated with PCa, the specific variation MET p.V378I, included in COSMIC database (COSM3411512), has not been extensively investigated. C-Met expression increases in advanced stages of the disease and more frequently in patients with bone metastases [24, 25]. In addition, c-MET protein expression is higher in poorly differentiated PCa with low PSA levels [26]. An inverse relationship between Androgen Receptor (AR) and c-MET has been already demonstrated [27] suggesting that a reduction of AR activity through androgen ablation may increase the expression of c-MET, directly contributing to androgen insensitivity and favoring tumor aggressiveness. Several clinical trials with different c-MET inhibitors (cabozantinib, tivantinib and other multi-target kinase inhibitors), have been recently performed [28–30] or are ongoing (NCT01428219, NCT01703065, NCT01630590, NCT01834651, NCT01812668, NCT01683994, NCT01519414), but the available data did not show any significant impact of these inhibitors on overall survival. These negative results could reflect the genetic variability highlighted in our study and the c-MET mutational status may potentially considered as a stratification factor in future studies aimed at evaluating the role c-MET inhibitors.

The genetic alterations in NOTCH1, IDH2, FGFR3 and STK11 more frequently reported in the LM risk group are located in regions that could influence protein functionality. Additional studies are needed to assess if GVs identified may deregulate or influence the corresponding gene expression/function. Retrospective studies showed a correlation between FGFR3 mutations and the development of good prognosis superficial bladder carcinoma [31, 32]. Interestingly in PCa FGFR3 mutations were associated with low-grade tumors. The F386L polymorphism has been reported in association with low-grade tumors and early disease stage [33] and in our series the FGFR3 p.F384L/p.F386L was associated with T<T2b patients (p=0.004).

Several studies on PCa have investigated the association between TMPRSS2:ERG fusion gene and outcome reporting conflicting results [34]. In our study, the presence of this rearrangement was correlated with the risk of recurrence, being the fusion gene mostly detected in patients with a low mutation rate. This observation suggests an involvement of TMPRSS2:ERG fusion gene in PCa aggressiveness independent from the gain of mutations. A previous study indicated that TMPRSS2-ERG gene fusion is a common event and occurs early in the development of invasive PCa [35]. Another study indicated that ERG gene alterations represent an initiating event that favors epithelial atypia and further progression to high-grade prostatic intraepithelial neoplasia and cancer [36].

After performing the DNA sequencing analyses, we also conducted an RNA sequencing analysis to determine gene expression of the 50 genes included in CHP2. We detected that FBXW7, GNAQ and JAK2 were over-expressed in the HI risk group without showing any correlation between gene expression and clinical-pathological characteristics. Additional investigation is needed to assess if these mRNA de-regulations may influence the protein expression and/or contribute to PCa development/progression.

We identified genetic markers potentially associated with disease pathogenesis, progression and response/resistance to treatment. However the translation of these results to the development of clinical diagnostic tests and to patient stratification in clinical trial is critical. Significant challenges, such as intra-tumoral heterogeneity and multifocality in primary tumors, limits the application of genomic medicine in prostate cancer and hinder the generation of risk stratification tools that correlate clinical outcome with the genomic landscape. Large clinical trials are needed to prospectively validate the utility of genetic markers for the clinical decision making.

In conclusion, this is one of the first retrospective studies that tested the feasibility of NGS for PCa genetic characterization using FFPE archival material. Although the limited number of patients limits the statistical power of the analyses, the reported results are in agreement with previously published data suggesting a complex mutational pattern in PCa. In addition, our data support the involvement of TP53, MET, FGFR3, CSF1R GVs in PCa, mainly influencing tumor progression or aggressiveness. Some of these GVs, such as CSFR1 SNPs or KIT p.M541L may potentially contribute, if data will be confirmed in larger studies, to customized treatments with targeted therapies.

## MATERIALS AND METHODS

### Patients and tissue samples

Sixty cases of localized or locally advanced PCa submitted to radical prostatectomy at San Luigi Gonzaga Hospital (Orbassano, Turin) between September 2003 and May 2009, with enough leftover FFPE tissue available and detailed clinical annotates were retrospectively extracted from an institutional clinical database. Based on PSA, GS and T, using the D'Amico criteria, tumors were categorized as high risk (HI, PSA>20 ng/ml, GS>7, T2c-T3, n=30) or low-intermediate risk (LM, low risk: PSA<10 ng/ml, GS <7, T1-T2a; intermediate risk: PSA: 10-20 ng/ml, GS=7, T2b, n=30).

Informed consent was previously obtained from each patient and the Institutional Review Board approved the study. All samples were de-identified and cases anonymized by a pathology staff member not involved in the study.

All samples were reviewed and classified according to UICC criteria [37].

### RNA isolation from paraffin-embedded tissues and quantitative real-time PCR

Serial 10 μm sections were cut in RNase-free conditions. The specimens were stained with hematoxylin-eosin, representative tumor areas were identified and isolated by means of microdissection using a scalpel at a magnification of 100x. RNA isolation was performed using commercially available RNA extraction kits for paraffin material according to the manufacturer's instructions (High Pure RNA paraffine kit; Roche Applied Science, Germany).

Complementary DNA was transcribed using random hexamer primers (Roche Applied Science) and M-MLV reverse transcriptase (200 U/ml; Invitrogen, Carlsbad; CA) according to standard protocols.

Expression levels for all investigated genes and an internal reference gene (β-actin) were assessed using a fluorescence-based real-time detection method (ABI PRISM 7900 Sequence Detection System–Taqman; Applied Biosystems, Foster City, CA,). Primers for β-actin and for androgen receptor have been previously reported [38, 39]. For androgen receptor and ERG:TMPRSS2 fusion gene TaqMan gene expression assays (AR: HS00171172_m1, TMPRSS2-ERG: HS003063375-ft) were diluted 1:20 with 1X Taqman Universal PCR Master Mix to a final volume of 20 μL (all reagents from Applied Biosystems). Cycling conditions were 50°C for 2 minutes and 95°C for 10 minutes followed by 46 cycles at 95°C for 15 seconds and 60°C for 1 minute. Relative gene expression levels were expressed as unit less ratios between 2 measurements (genes of interest/internal reference gene). Total RNAs (Stratagene, La Jolla, CA) were used as control calibrators on each plate.

### Genomic DNA/total mRNA extraction and NGS

Nucleic acids for NGS were obtained from tissues after manual microdissection with enrichment for at least 50% of neoplastic cells. Genomic DNA (gDNA) and total mRNA were extracted using AllPrep DNA/RNA FFPE Kit (Qiagen, Hilden, Germany) following the manufacturer's instructions. gDNA and mRNA were quantified using fluorometer Qubit platform (Invitrogen, Carlsbad, CA).

NGS analyses were performed on the Ion Torrent Personal Genome Machine (PGM, Life Technologies, Grand Island, USA). Tumor samples were tested using a commercially available library kit (gDNA: Ion AmpliSeq Cancer Hotspot Panel v.2 (CHP2), mRNA: Ion AmpliSeq RNA Cancer Panel) to investigate 50 cancer-associated genes (See Supplementary Table S1). Each amplicon library was generated starting from 10 ng of gDNA or mRNA, as indicated by the manufacturer, and barcoded with Ion Xpress Barcode Adaptors Kit (Life Technologies). DNA Library quantification was performed using the PCR quantification kit and the 7900HT real time PCR system (Life Technologies), diluted in nuclease-free water to obtain a final concentration of 100 pM. Emulsion PCR was performed on Ion PGM™ Template One Touch 2 system (Life Technologies). The quality of the emulsion PCRs was measured using the Qubit IonSphere Quality control kit (Life Technologies). IonSphere Particles with DNA were isolated and sequenced on Ion 316 Chip using the Ion PGM™ Sequencing 200 Kit (Life Technologies). Only sample sequences with at least a quality score of AQ20 (1 misaligned base per 100 bases) were considered for further analyses.

#### DNA target sequencing

The inclusion criteria for the analyses mainly considered the coverage target for each sample and this value was set at a minimum average deep of 100 reads for each amplicon (whole coverage: min=18, max=3875, amplicons average=1190). Variant Caller plugin included in Torrent Suite Software v.4.2.1 was used to identify variations in target regions and genetic annotation was performed with Annovar software [40] (COSMIC database v.70, SNPs database v.138). Each of the identified genetic variation was coded according to “plus strand” of Human Genome assembly hg19. Some of the commonest genetic variations identified in the study were further validated and specific primers listed in CHP2 supplementary files were used for PCR. The quality of the resulting amplicons was checked by LabChip® GX (PerkinElmer, Waltham, MA, USA) and subsequently validated by Sanger sequencing. For the genetic variations in study, the correlation between Sanger and NGS was always ≥ 80%.

#### RNA target sequencing

AmpliSeqRNA plugin included in Torrent Suite Software v.4.2.1 was used to normalize expression levels of each amplicon in the RNA-sequencing analysis. Differentially expressed/mutated genes between the HI and LM risk groups were identified using the RNA-seq version of Significance Analysis of Microarray (SAMseq) included in the R package “samr”. The significant differential expression levels were subsequently tested in qPCR analysis.

### Statistical analysis

Statistical correlation between gene variations with allelic frequency (AF) ≥10% and clinical-pathological features were investigated by Fisher exact test.

Time to progression (TTP) was defined as the time from the prostatectomy until the first evidence of disease progression, defined as confirmed PSA increase above 0.2 ng /ml. The log rank test was used to assess differences between groups. The Cox proportional hazards regression model was performed to analyze independent prognostic factors and TTP. Only the variables that were found to be significant in the univariate analyses (p<0.05) were entered into the multivariable analysis to determine the most significant factors for predicting disease outcome. Statistical analysis was elaborated using R statistical software [41].

## SUPPLEMENTARY FIGURES AND TABLES




