# Supplementary material for: Retrospective study testing next generation sequencing of selected cancer-associated genes in resected prostate cancer
Source: Oncotarget. 2016 Feb 12;7(12):14394–404. doi: 10.18632/oncotarget.7343 (PMC4924723; doi:10.18632/oncotarget.7343)
Supplement: Supplementary file 1 [file oncotarget-07-14394-s001.pdf]

SUPPLEMENTARY TABLE

Supplementary Table 1: CHP2 genetic profile in the whole cohort, high risk patients and low-intermediate risk patients

See Supplementary File 1
